# Supplementary material for: Molecular Detection and Genetic Characterization of Potential Zoonotic Swine Enteric Viruses in Northern China
Source: Pathogens. 2022 Mar 30;11(4):417. doi: 10.3390/pathogens11040417 (PMC9031704; doi:10.3390/pathogens11040417)
Supplement: Supplementary file 1 [file pathogens-11-00417-s001.zip › EMCV.pdf]

>EMCV-1|SPF
GTGAGAGCAAGCCTCGCAAAGACAGGATATAAGATAACTCCCGCTAACAAAACTTCTACCTTTCCTCTTAAT
TCGACGC
TTGAAGACGTTGTCTTCTTAAAAAGAAAGTTTAAGAAAGAGGGCCCTCTGTATCGGCCTGTCATGAACAGAG
AGGCGTT
GGAAGCAATGTTGTCATACTATCGTCCAGGGACTCTATCTGAGAAACTCACTTCGATCACTATGCTTGCC
>Emcv-2|SPF
GTGAGAGCAAGCCTCGCAAAGACAGGATATAAGATAACTCCCGCTAACAAAACTTCTACCTTTCCTCTTAAT
TCGACGC
TTGAAGACGTTGTCTTCTTAAAAAGAAAGTTTAAGAAAGAGGGCCCTCTGTATCGGCCTGTCATGAACAGAG
AGGCGTT
GGAAGCAATGTTGTCATACTATCGTCCAGGGACTCTATCTGAGAAACTCACTTCGATCACTATGCTTGCC
>EMCV-3
GTGAGAGCAAGCCTCGCAAAGACAGGATATAAGATAACTCCCGCTAACAAAACTTCTACCTTTCCTCTTAAT
TCGACGC
TTGAAGACGTTGTCTTCTTAAAAAGAAAGTTTAAGAAAGAGGGCCCTCTGTATCGGCCTGTCATGAACAGAG
AGGCGTT
GGAAGCAATGTTGTCATACTATCGTCCAGGGACTCTATCTGAGAAACTCACTTCGATCACTATGCTTGCC
>EMCV-4|SPF
GTGAGAGCAAGCCTCGCAAAGACAGGATATAAGATAACTCCCGCTAACAAAACTTCTACCTTTCCTCTTAAT
TCGACGC
TTGAAGACGTTGTCTTCTTAAAAAGAAAGTTTAAGAAAGAGGGCCCTCTGTATCGGCCTGTCATGAACAGAG
AGGCGTT
GGAAGCAATGTTGTCATACTATCGTCCAGGGACTCTATCTGAGAAACTCACTTCGATCACTATGCTTGCC
>EMCV-5
GTGAGAGCAAGCCTCGCAAAGACAGGATATAAGATAACTCCCGCTAACAAAACTTCTACCTTTCCTCTTAAT
TCGACGC
TTGAAGACGTTGTCTTCTTAAAAAGAAAGTTTAAGAAAGAGGGCCCTCTGTATCGGCCTGTCATGAACAGAG
AGGCGTT
GGAAGCAATGTTGTCATACTATCGTCCAGGGACTCTATCTGAGAAACTCACTTCGATCACTATGCTTGCC
>EMCV-6|SPF
GTGAGAGCAAGCCTCGCAAAGACAGGATATAAGATAACTCCCGCTAACAAAACTTCTACCTTTCCTCTTAAT
TCGACGC
TTGAAGACGTTGTCTTCTTAAAAAGAAAGTTTAAGAAAGAGGGCCCTCTGTATCGGCCTGTCATGAACAGAG
AGGCGTT
GGAAGCAATGTTGTCATACTATCGTCCAGGGACTCTATCTGAGAAACTCACTTCGATCACTATGCTTGCC
>EMCV-7
GTGAGAGCAAGCCTCGCAAAGACAGGATATAAGATAACTCCCGCTAACAAAACTTCTACCTTTCCTCTTAAT
TCGACGC
TTGAAGACGTTGTCTTCTTAAAAAGAAAGTTTAAGAAAGAGGGCCCTCTGTATCGGCCTGTCATGAACAGAG
AGGCGTT
GGAAGCAATGTTGTCATACTATCGTCCAGGGACTCTATCTGAGAAACTCACTTCGATCACTATGCTTGCC
>EMCV-8
GTGAGAGCAAGCCTCGCAAAGACAGGATATAAGATAACTCCCGCTAACAAAACTTCTACCTTTCCTCTTAAT
TCGACGC
TTGAAGACGTTGTCTTCTTAAAAAGAAAGTTTAAGAAAGAGGGCCCTCTGTATCGGCCTGTCATGAACAGAG
AGGCGTT
GGAAGCAATGTTGTCATACTATCGTCCAGGGACTCTATCTGAGAAACTCACTTCGATCACTATGCTTGCC
>EMCV-9
GTGAGAGCAAGCCTCGCAAAGACAGGATATAAGATAACTCCCGCTAACAAAACTTCTACCTTTCCTCTTAAT
TCGACGC
TTGAAGACGTTGTCTTCTTAAAAAGAAAGTTTAAGAAAGAGGGCCCTCTGTATCGGCCTGTCATGAACAGAG
AGGCGTT

GGAAGCAATGTTGTCATACTATCGTCCAGGGACTCTATCTGAGAAACTCACTTCGATCACTATGCTTGCC
>EMCV-10
GTGAGAGCAAGCCTCGCAAAGACAGGATATAAGATAACTCCCGCTAACAAAACTTCTACCTTTCCTCTTAAT
TCGACGC
TTGAAGACGTTGTCTTCTTAAAAAGAAAGTTTAAGAAAGAGGGCCCTCTGTATCGGCCTGTCATGAACAGAG
AGGCGTT
GGAAGCAATGTTGTCATACTATCGTCCAGGGACTCTATCTGAGAAACTCACTTCGATCACTATGCTTGCC
>EMCV-11
GTGAGAGCAAGCCTCGCAAAGACAGGATATAAGATAACTCCCGCTAACAAAACTTCTACCTTTCCTCTTAAT
TCGACGC
TTGAAGACGTTGTCTTCTTAAAAAGAAAGTTTAAGAAAGAGGGCCCTCTGTATCGGCCTGTCATGAACAGAG
AGGCGTT
GGAAGCAATGTTGTCATACTATCGTCCAGGGACTCTATCTGAGAAACTCACTTCGATCACTATGCTTGC
