# Supplementary material for: Molecular Detection and Genetic Characterization of Potential Zoonotic Swine Enteric Viruses in Northern China
Source: Pathogens. 2022 Mar 30;11(4):417. doi: 10.3390/pathogens11040417 (PMC9031704; doi:10.3390/pathogens11040417)
Supplement: Supplementary file 1 [file pathogens-11-00417-s001.zip › PAstV.pdf]

>PAstro-1|SPF
AACTCTATGCTGGACTTGTAACACCTGCAAGAAAGCTCCCTAATGTCACAGCGCTGCATG
GGAAACTCCTGAGCCTACAACTCTTGATGCACAACCATCCTGACAGTGCCTTTAAAGACT
ACATCAATAAATGTTTGGCTGAAACAGCGAGGCACGCCGAGGACCTGCCTGCAAGACTCA
CAGAAAGGCAGATGGACAGGCTTTGGAGGGGCGGACCAAAGCATAAGCCTAATGGCTAAC
AACCAGAAAAATGTCCAACCCAAGGTGGTTACGACTACTACGACGACTACTAGTCGTCGT
GGCGGGCGTCGCCGCCGGCGGAATCCTCGCGCATCTACATCTAGTAAGACCACTGTTAGG
AAGGTGGCCGTTCTTGGACGCTCTCGTAGGTTTCCTCGTCGGCGCACTACTAGGACTGGT
AATCTTCCTAAGCCAAATAATTCAATGTTCAGGCAAAAAATAACAGCAACATTAGGCTCT
GTGGGAGCTAATAAAGGAAGTGGTATAGAATTAGAAATGTCAGCACTAATTAATCCAGCA
TTGATGAAGGAGACAACAGGGTCTAATCAGTTTGGACCCTTACAAATTGCAGCATCAACC
TATAACTTTTGGAGGGTAGATTATATAGATATAAAATTGACACCTTTAGTTGGTGCATCT
GCAGTGTCAGGGACCATGGTTAGAACATCTTTAAATCTGGCAGCACAACCTGGGAACGTA
TCATGGT
>PAstro-2|SPF
AACTCTATGCTGGACTTGTAACACCTGCAAGAAAGCTCCCTAATGTCACAGCGCTGCATG
GGAAACTCCTGAGCCTACAACTCTTGATGCACAACCATCCTGACAGTGCCTTTAAAGACT
ACATCAATAAATGTTTGGCTGAAACAGCGAGGCACGCCGAGGACCTGCCTGCAAGACTCA
CAGAAAGGCAGATGGACAGGCTTTGGAGGGGCGGACCAAAGCATAAGCCTAATGGCTAAC
AACCAGAAAAATGTCCAACCCAAGGTGGTTACGACTACTACGACGACTACTAGTCGTCGT
GGCGGGCGTCGCCGCCGGCGGAATCCTCGCGCATCTACATCTAGTAAGACCACTGTTAGG
AAGGTGGCCGTTCTTGGACGCTCTCGTAGGTTTCCTCGTCGGCGCACTACTAGGACTGGT
AATCTTCCTAAGCCAAATAATTCAATGTTCAGGCAAAAAATAACAGCAACATTAGGCTCT
GTGGGAGCTAATAAAGGAAGTGGTATAGAATTAGAAATGTCAGCACTAATTAATCCAGCA
TTGATGAAGGAGACAACAGGGTCTAATCAGTTTGGACCCTTACAAATTGCAGCATCAACC
TATAACTTTTGGAGGGTAGATTATATAGATATAAAATTGACACCTCTAGTTGGTGCATCT
GCAGTGTCAGGGACCATGGTTAGAACATCTTTAAATCTGGCAGCACAACCTGGGAACGTA
TCATGGT
>PAstro-3|SPF
AACTCTATGCTGGACTTGTAACACCTGCAAGAAAGCTCCCTGATGCCACAGCGCTGCATG
GGAAACTCCTGAGCCTACAACTCCTGATGCATAACCATCCTGACAGTGCCTTTAAAGATT
ACATCAATAAATGCTTGGCTGAAACAGCGAGGCACGCCGAGGATCTGCCTGCAAGACTTA
CAGAAAGGCAGATGGACAGGCTTTGGAGGGGCGGACCAAAGCATAAGCCTAATGGCTAAC
AACCAGAAAAATGTCCAACCCAAGGTGGTTACGACTACTACGACGACTACTAGTCGTCGT
GGCGGGCGTCGCCGCCGGCGGAATCCTCGCGCATCTACATCTAGTAAGACCACTGTTAGG
AAGGTGGCCGTTCTTGGACGCTCTCGTAGGTTTCCTCGTCGGCGCACTACTAGGACTGGT
AATCTTCCTAAGCCAAATAATTCAATGTTCAGGCAAAAAATAACAGCAACATTAGGCTCT
GTGGGAGCTAATAAAGGAAGTGGTATAGAATTAGAAATGTCAGCACTAATTAATCCAGCA
TTGATGAAGGAGACAACAGGGTCTAATCAGTTTGGACCCTTACAAATTGCAGCATCAACC
TATAACTTTTGGAGGGTAGATTATATAGATATAAAATTGACACCTTTAGTTGGTGCATCT
GCAGTGTCAGGGACCATGGTTAGAACATCTTTAAATCTGGCAGCACAACCTGGGAACGCA
TCATGGT
>PAstro-4|SPF
AACTCTATGCTGGACTTGTAACACCTGCAAGAAAGCTCCCTAATGTCACAGCGCTGCATG
GGAAACTCCTGAGCCTACAACTCTTGATGCACAACCATCCTGACAGTGCCTTTAAAGACT
ACATCAATAAATGTTTGGCTGAAACAGCGAGGCACGCCGAGGACCTGCCTGCAAGACTCA
CAGAAAGGCAGATGGACAGGCTTTGGAGGGGCGGACCAAAGCATAAGCCTAATGGCTAAC
AACCAGAAAAATGTCCAACCCAAGGTGGTTACGACTACTACGACGACTACTAGTCGTCGT
GGCGGGCGTCGCCGCCGGCGGAATCCTCGCGCATCTACATCTAGTAAGACCACTGTTAGG
AAGGTGGCCGTTCTTGGACGCTCTCGTAGGTTTCCTCGTCGGCGCACTACTAGGACTGGT
AATCTTCCTAAGCCAAATAATTCAATGTTCAGGCAAAAAATAACAGCAACATTAGGCTCT
GTGGGAGCTAATAAAGGAAGTGGTATAGAATTAGAAATGTCAGCACTAATTAATCCAGCA
TTGATGAAGGAGACAACAGGGTCTAATCAGTTTGGACCCTTACAAATTGCAGCATCAACC

TATAACTTTTGGAGGGTAGATTATATAGATATAAAATTGACACCTTTAGTTGGTGCATCT
GCAGTGTCAGGGACCATGGTTAGAACATCTTTAAATCTGGCAGCACAACCTGGGAACGTA
TCATGGT
>PAstro-5|SPF
AACTCTATGCTGGACTTGTAACACCTGCAAGAAAGCTCCCTGATGTTACAGCGCTGCATG
GGAAACTCCTGAGCCTACAACTCCTGATGCATAACCATCCTGACAGTGCCTTCAAGAACT
ACATTGATAAATGTTTGGCTGAAACAGCGAGGCACGCCGAGGATCTGCCTGCAAGACTCA
CAGAAAGGCAGATGGATAGGCTTTGGAGGGGCGGACCAAAGCATAAGCCTAATGGCTAAT
ACTAAGAACAATGTCCAGCCTCAGGTGGTCACGACTACTACGACGACTACTAGTCGTCGC
GGTAGGCGTCGCCGCAGGCGCGCTACTCGCCCATCTCAGCCTACTACAACCACTGTTAGG
AGGGTTACCACTGCTAGGCAGGCTACTCGGCCTCGCCGTGGGCGCCGTAATCGCTTTGGC
GCTCCTGGTAAATCGGCTCCTAGCAGCATTAGACAAAGAATCACAGCCACATTAGGGACA
GTTGGGTCCAACCAGGGTAATGACATAGAAATGGAGATGGCCTGTCTTCTAAACCCAGCA
CTAATGAAGGAAACAACAGGTTCAAATCAGCATGGACCTCTTCAAATCTATGCCTCAACA
TACAGTCTCTGGAGGATTGAAAGGATCATACTTAAGTTGACACCTCTTGTTGGCCCATCT
GCAGTATCAGGTACAGCTGTTAGAGCATCATATAATCCATCTGGCCAGCCAGGCTCCCCA
TCTTGGT
>PAstro-6
AACTCTATGCTGGACTTGTGACACCTGCAAGAAAGCTCCCTGATGTCACAGCGCTGCATG
GGAAACTCCTGAGCCTACAACTCTTGATGCATAACCATCCTGACAGTGCCTTTAAAGATT
ACATCAATAAATGTTTGGCTGAAACAGCGAGGCACGCCGAGGATCTGCCTGCAAGACTCA
CAGAAAGGCAGATGGACAGGCTTTGGAGGGGCGGACCAAAGCATAAGCCTAATGGCTAAC
AACCAGAAAAATGTCCAACCCAAGGTGGTTACGACTACTACGACGACTACTAGTCGTCGT
GGTGGGCGTCGCCGCCGGCGGACTCCTCGCCCATCTACAGCTAATACAACCACTGTTAGG
AAGGTGACCATTCTTGGACAATCTCGTAGGTTTCCTCGTCGGCGCCCTACTAGGACTGGT
AATCCTCCTCAACCAGATACTTCCATGTTCAGACAAAAAATAACAGCAACATTAGGCTCT
GTGGGATCTAATAAAGGAGATGGTATTGAACTAGAGATGGCAGCACTTATGAATCCAGCA
TTGATGAAGGAGACAACAGGGTCTAATCAATTTGGACCCTTACAAATGGCAGCATCAAAC
TATAACATGTGGAGAGTAGATTATATATATATAAAATTGACACCTCTAGTTGGTGCCTCA
GCAGTGTCTGGAACCATGGTCAGATCCTCTTTAAATCTGGCAGCACCACCTGGCTCCGCA
TCATGGT
>PAstro-7
AACTCTATGCTGGACTTGTGACACCTGCAAGAAAGCTTCCTGATGCCATAGCGCTGCATG
GGAAACTCCTGAGCCTACAACTTCTGATGCATAACCATCCTGACAGTGCCTTTAAAGATT
ACATCAACAAATGTTTGGCTGAAACAGCGAGGCACGCCGAGGATCTGCCTGCAAGACTCA
CAGAAAGGCAGATGGACAGGCTTTGGAGGGGCGGACCAAAGCATAAGCCTAATGGCTAAC
AACCAGAAAAATGTCCAACCCACTGTGGTTACGACTACTACGACGATTGTTAACCGTCGT
GGTAGGCGTCGCCGCCGGCGTACTCCTCGCCCATCTCAGGCTAATACAACCACTGTTAGG
AAGGTTACCATTATTAGACAATCTGCTAGGGTTCCTCGTAGGCGCCGTACTCGGACTGGT
AATCCTAATCAATCAGCACCTCCCATGTTCAGACAAAAAATAACAGCAACTTTGGGCTCT
GTGGGATCTAATCAAGGAGATGCCATTGAACTGGAGATGGCTGCACTTCTGAATCCAGCA
TTGACTAAGGAGACAACAGGCTCTAACCAATTTGGACCTTTACAGATGTGGGCTGCAAAC
TATAACATGTGGAGGGTAGAAAACATCTAAATAAAATTTGCACCTCTAGTTGGTGCCTCT
GCAGTGTCTGGAACCATGATCAGATGCTCATTAAATTTGGCAGCCCCACCTGGCTACGCA
TCATGGT
>PAstro-8
AACTCTATGCTGGACTTGTGACACCTGCAAGAAAGCTTCCTGATGTCATAGCGCTGCATG
GGAAACTCCTGAGCCTACAACTTCTGATGCATAACCATCCTGACAGTGCCTTTAAAGATT
ACATCAACAAATGTTTGGCTGAAACAGCGAGGCACGCCGAGGATCTGCCTGCAAGACTCA
CAGAAAGGCAGATGGACAGGCTTTGGAGGGGCGGACCAAAGCATAAGCCTAATGGCTAAC
AACCAGAAAAATGTCCAACCCACTGTGGTCACGACTACTACGACGACTGTTAACCGTCGT
GGTAGGCGTCGCCGCAGGCGTGCTAATCGCCCATCTCAAGCTAATACAACCACTGTTAGG
AAGGTTACCACTACTAGACAATCTGCTAGGGCTCCTCGTCGGCGCCGTAATCGCACTGGT

AATCCTAATCAATCGGCTCCTCCCATCTTCAGACAAAAAATAACAGCAACTTTGGGCACT
GTTGGATCAAATCAAGGAGATGCCATTGAACTGGAGATGGCTGCACTTCTGAATCCAGCA
TTGACTAAGGAGACAACAGGCTCTAATCAATTTGGACCTTTACAGATGTCGGCTGCCAAC
TATAATATGTGGAAGGTAGATAATATCTATATAAAATTGATACCTCTTGTTGGTGCCTCA
GCAGTTTCTGGAACAGTGATCAGATGCTCATTAAATTTAGCAGCAACACCTGGCTCCGCA
TCATGGT
>PAstro-9
AACTCTATGCTGGACTTGTAACACCTGCAAGAAAGCTTCCTGATGTCACAGCGCTGCATG
GGAAACTCCTGAGCCTACAACTCCTGATGCATAACCATCCTGACAGTGCCTTCAAAGATT
ACATCAACAAATGTTTGGCTGAAACAGCGAGGCACGCTGAGGATCTGCCTGCAAGACTTA
CAAAAAGGCAGATGGACAGGCTTTGGAGGGGCGGACCAAAGCATAAGCCTAATGGCTAAC
ACTAAAAACAATGTCCAACCCCAAGTGGTCACGACTACTACGACGACTACTATTCGTCGC
GGTAGGCGTCGCCGCAGGCGCGCTAATCCCACATCTCAAACTGCTACAACCACTGTTAGG
AAAGTTACCACTGCTAGACAATCTAATAGGCTTCCCCGTCGGCGCCGTAATCGCTTTGGC
GCTGCTGGTCAACCGGGTGCTTCCATCTTCAAACACAGAATCACAGCAACATTAGGTACA
ATTGGTTCAAATGAAGGTGATAAAATTGAACTAGAGATGGCCGCACTTTTGAACCCCGCA
CTCATGAAGGAAACAACTGGGTCAAATCAATATGGACCTTTACAAATGTATGCATCAAAC
TATAATCTTTGGAGAGTCAACAATATCATTTTAAAATTGATACCCCTTGTTGGTGGATCA
GCTGTCTCTGGCACTGCTGTCCGGGCTTCTCTTAACCTAGCAGGAACACCTGGGATCAGC
ATCATGGT
>PAstro-10
AACTCTATGCTGGACTTGTAACACCTGCAAGAAAGCTCCCTGATGTTACAGCGCTGCATG
GGAAACTCCTGAGCCTACAACTCCTGATGCATAACCATCCTGACAGTGCCTTCAAGAACT
ACATTGATAAATGTTTGGCTGAAACAGCGAGGCACGCCGAGGATCTGCCTGCAAGACTCA
CAGAAAGGCAGATGGATAGGCTTTGGAGGGGCGGACCAAAGCATAAGCCTAATGGCTAAT
ACTAAGAACAATGTCCAGCCTCAGGTGGTCACGACTACTACGACGACTACTAGTCGTCGC
GGTAGGCGTCGCCGCAGGCGCGCTACTCGCCCATCTCAGCCTACTACAACCACTGTTAGG
AGGGTTACCACTGCTAGGCAGGCTACTCGGCCTCGCCGTGGGCGCCGTAATCGCTTTGGC
GCTCCTGGTAAATCGGCTCCTAGCAGCATTAGACAAAGAATCACAGCCACATTAGGGACA
GTTGGGTCCAACCAGGGTAATGACATAGAAATGGAGATGGCCTGTCTTCTAAACCCAGCA
CTAATGAAGGAAACAACAGGTTCAAATCAGCATGGACCTCTTCAAATCTATGCCTCAACA
TACAGTCTCTGGAGGATTGAAAGGATCATACTTAAGTTGACACCTCTTGTTGGCCCATCT
GCAGTATCAGGTACAGCTGTTAGAGCATCATATAATCCATCTGGCCAGCCAGGCTCCCCA
TCTTGGT
>PAstro-11
AACTCTATGCTGGACTTGTAACACCTGCAAGAAAGCTCCCTAATGTCACAGCGCTGCATG
GGAAACTCCTGAGCCTACAACTCTTGATGCACAACCATCCTGACAGTGCCTTTAAAGACT
ACATCAATAAATGTTTGGCTGAAACAGCGAGGCACGCCGAGGACCTGCCTGCAAGACTCA
CAGAAAGGCAGATGGACAGGCTTTGGAGGGGCGGACCAAAGCATAAGCCTAATGGCTAAC
AACCAGAAAAATGTCCAACCCAAGGTGGTTACGACTACTACGACGACTACTAGTCGTCGT
GGCGGGCGTCGCCGCCGGCGGAATCCTCGCGCATCTACATCTAGTAAGACCACTGTTAGG
AAGGTGGCCGTTCTTGGACGCTCTCGTAGGTTTCCTCGTCGGCGCACTACTAGGACTGGT
AATCTTCCTAAGCCAAATAATTCAATGTTCAGGCAAAAAATAACAGCAACATTAGGCTCT
GTGGGAGCTAATAAAGGAAGTGGTATAGAATTAGAAATGTCAGCACTAATTAATCCAGCA
TTGATGAAGGAGACAACAGGGTCTAATCAGTTTGGACCCTTACAAATTGCAGCATCAACC
TATAACTTTTGGAGGGTAGATTATATAGATATAAAATTGACACCTCTAGTTGGTGCATCT
GCAGTGTCAGGGACCATGGTTAGAACATCTTTAAATCTGGCAGCACAACCTGGGAACGTA
TCATGGT
>PAstro-12
AACTCTATGCTGGACTTGTAACACCTGCAAGAAAGCTCCCTGATGCCACAGCGCTGCATG
GGAAACTCCTGAGCCTACAACTCCTGATGCATAACCATCCTGACAGTGCCTTTAAAGATT
ACATCAATAAATGCTTGGCTGAAACAGCGAGGCACGCCGAGGATCTGCCTGCAAGACTTA
CAGAAAGGCAGATGGACAGGCTTTGGAGGGGCGGACCAAAGCATAAGCCTAATGGCTAAC

AACCAGAAAAATGTCCAACCCAAGGTGGTTACGACTACTACGACGACTACTAGTCGTCGT
GGCGGGCGTCGCCGCCGGCGGAATCCTCGCGCATCTACATCTAGTAAGACCACTGTTAGG
AAGGTGGCCGTTCTTGGACGCTCTCGTAGGTTTCCTCGTCGGCGCACTACTAGGACTGGT
AATCTTCCTAAGCCAAATAATTCAATGTTCAGGCAAAAAATAACAGCAACATTAGGCTCT
GTGGGAGCTAATAAAGGAAGTGGTATAGAATTAGAAATGTCAGCACTAATTAATCCAGCA
TTGATGAAGGAGACAACAGGGTCTAATCAGTTTGGACCCTTACAAATTGCAGCATCAACC
TATAACTTTTGGAGGGTAGATTATATAGATATAAAATTGACACCTTTAGTTGGTGCATCT
GCAGTGTCAGGGACCATGGTTAGAACATCTTTAAATCTGGCAGCACAACCTGGGAACGCA
TCATGGT
