# Supplementary material for: Molecular Detection and Genetic Characterization of Potential Zoonotic Swine Enteric Viruses in Northern China
Source: Pathogens. 2022 Mar 30;11(4):417. doi: 10.3390/pathogens11040417 (PMC9031704; doi:10.3390/pathogens11040417)
Supplement: Supplementary file 1 [file pathogens-11-00417-s001.zip › PKV.pdf]

>PKV1|SPF
GTCGAGAAACATCTCGAACGTCTCATCGGAGACGAACGGGTGACCCGCTACATCGAGACC
ATACGACACTCGCGCCATGTCTTTGGCAACGAGACGTATGAGATGATTGGCGGTAACCCA
TCTGGATGCGTTGGCACCTCTATCATCAACACCATCATCAACAACATTTGTGTCCTCTCT
GCCCTTATCCAACACCCAGACTTTTCTCCTGAATCCTTCAGAATTCTGGCCTATGGTGAT
GATGTGATCTATGGGTGTGATCCGCCCATTCATCCATCATTCATCAAGGAG
>PKV2|SPF
GTCGAGAAACATCTCGAACGTCTCATCGGAGACGAACGGGTGACCCGCTACATCGAGACC
ATACGACACTCGCGCCATGTCTTTGGCAACGAGACGTATGAGATGATTGGCGGTAACCCA
TCTGGATGCGTTGGCACTTCTATCATCAACACCATCATCAACAACATTTGTGTCCTCTCT
GCCCTTATCCAACACCCTGACTTCTCTCCTGAATCCTTCAGAATTCTGGCCTATGGTGAT
GATGTGATCTATGGGTGTGACCCGCCCATTCATCCATCATTCATCAAGGAG
>PKV3|SPF
GTCGAGAAACATCTCGAACGTCTCATCGGAGACGAACGGGTGACCCGCTACATCGAGACC
ATACGACACTCGCGCCATGTCTTTGGCAACGAGACGTATGAGATGATTGGCGGTAACCCA
TCTGGATGCGTTGGCACCTCTATCATCAACACCATCATCAACAACATTTGTGTCCTCTCT
GCCCTTATCCAACACCCAGACTTTTCTCCTGAATCCTTCAGAATTCTGGCCTATGGTGAT
GATGTGATCTATGGGTGTGATCCGCCCATTCATCCATCATTCATCAAGGAG
>PKV4|SPF
GTTGAGAAACATCTCGAACGTCTCACTGGAGACGAGCGGGTGACCCGCTATATCGAGACC
ATACGCCACTCGCGCCATGTCTTTGGTAGCGAGACGTATGAGATGATTGGCGGCAACCCG
TCTGGATGCGTTGGTACTTCCATCATCAACACCATCATCAACAACATTTGTGTCCTCTCT
GCCCTCATTCAGCACCCTGATTTCTCTCCTGAATCCTTCAGAATTTTGGCCTACGGTGAT
GACGTGATCTATGGGTGCGACCCACCCATCCATCCGTCATTCATCAAGGAG
>PKV5|SPF
GTTGAGAAACATCTCGAACGTCTCACTGGAGACGAGCGGGTGACCCGCTATATCGAGACC
ATACGCCACTCGCGCCATGTCTTTGGTAGCGAGACGTATGAGATGATTGGCGGCAACCCG
TCTGGATGCGTTGGTACTTCCATCATCAACACCATCATCAACAACATTTGTGTCCTCTCT
GCCCTCATTCAGCACCCTGATTTCTCTCCTGAATCCTTCAGAATTTTGGCCTACGGTGAT
GACGTGATCTATGGGTGCGACCCACCCATCCATCCGTCATTCATCAAGGAG
>PKV6
GTTGAGAAACATCTCGAACGTCTCACTGGAGACGAGCGGGTGACCCGCTATATCGAGACC
ATACGCCACTCGCGCCATGTCTTTGGTAGCGAGACGTATGAGATGATTGGCGGCAACCCG
TCTGGATGCGTTGGTACTTCCATCATCAACACCATCATCAACAACATTTGTGTCCTCTCT
GCCCTCATTCAGCACCCTGATTTCTCTCCTGAATCCTTCAGAATTTTGGCCTACGGTGAT
GACGTGATCTATGGGTGCGACCCACCCATCCATCCGTCATTCATCAAGGAG
>PKV7
GTTGAGAAACATCTCGAACGTCTCACTGGAGACGAGCGGGTGACCCGCTATATCGAGACC
ATACGCCACTCGCGCCATGTCTTTGGTAGCGAGACGTATGAGATGATTGGCGGCAACCCG
TCTGGATGCGTTGGTACTTCCATCATCAACACCATCATCAACAACATTTGTGTCCTCTCT
GCCCTCATTCAGCACCCTGATTTCTCTCCTGAATCCTTCAGAATTTTGGCCTACGGTGAT
GACGTGATCTATGGGTGCGACCCACCCATCCATCCGTCATTCATCAAGGAG
>PKV8
GTTGAGAAACATCTCGAACGTCTCACTGGAGACGAGCGGGTGACCCGCTATATCGAGACC
ATACGCCACTCGCGCCATGTCTTTGGTAGCGAGACGTATGAGATGATTGGCGGCAACCCG
TCTGGATGCGTTGGTACTTCCATCATCAACACCATCATCAACAACATTTGTGTCCTCTCT
GCCCTCATTCAGCACCCTGATTTCTCTCCTGAATCCTTCAGAATTTTGGCCTACGGTGAT
GACGTGATCTATGGGTGCGACCCACCCATCCATCCGTCATTCATCAAGGAG
>PKV9
GTTGAGAAACATCTCGAACGTCTCACTGGAGACGAGCGGGTGACCCGCTATATCGAGACC
ATACGCCACTCGCGCCATGTCTTTGGTAGCGAGACGTATGAGATGATTGGCGGCAACCCG
TCTGGATGCGTTGGTACTTCCATCATCAACACCATCATCAACAACATTTGTGTCCTCTCT
GCCCTCATTCAGCACCCTGATTTCTCTCCTGAATCCTTCAGAATTTTGGCCTACGGTGAT

GACGTGATCTATGGGTGCGACCCACCCATCCATCCGTCATTCATCAAGGAG
>PKV10
GTTGAGAAACATCTCGAACGTCTCACTGGAGACGAGCGGGTGACCCGCTATATCGAGACC
ATACGCCACTCGCACCATGTCTTTGGTAGCGAGACGTATGAGATGATTGGCGGCAACCCG
TCTGGATGCGTTGGTACTTCCATCATCAACACCATCATCAACAACATTTGTGTCCTCTCT
GCCCTCATTCAGCACCCTGATTTCTCTCCTGAATCCTTCAGAATTTTGGCCTACGGTGAT
GACGTGATCTATGGGTGCGACCCACCCATCCATCCGTCATTCATCAAGGAG
>PKV11
GTTGAGAAACATCTCGAACGTCTCACTGGAGACGAGCGGGTGACCCGCTATATCGAGACC
ATACGCCACTCGCGCCATGTCTTTGGTAGCGAGACGTATGAGATGATTGGCGGCAACCCG
TCTGGATGCGTTGGTACTTCCATCATCAACACCATCATCAACAACATTTGTGTCCTCTCT
GCCCTCATTCAGCACCCTGATTTCTCTCCTGAATCCTTCAGAATTTTGGCCTACGGTGAT
GACGTGATCTATGGGTGCGACCCACCCATCCATCCGTCATTCATCAAGGAG
>PKV12
GTTGAGAAACATCTCGAACGTCTCACTGGAGACGAGCGGGTGACCCGCTATATCGAGACC
ATACGCCACTCGCGCCATGTCTTTGGTAGCGAGACGTATGAGATGATTGGCGGCAACCCG
TCTGGATGCGTTGGTACTTCCATCATCAACACCATCATCAACAACATTTGTGTCCTCTCT
GCCCTCATTCAGCACCCTGATTTCTCTCCTGAATCCTTCAGAATTTTGGCCTACGGTGAT
GACGTGATCTATGGGTGCGACCCACCCATCCATCCGTCATTCATCAAGGAG
>PKV13
GTTGAGAAACATCTCGAACGTCTCACTGGAGACGAGCGGGTGACCCGCTATATCGAGACC
ATACGCCACTCGCGCCATGTCTTTGGTAGCGAGACGTATGAGATGATTGGCGGCAACCCG
TCTGGATGCGTTGGTACTTCCATCATCAACACCATCATCAACAACATTTGTGTCCTCTCT
GCCCTCATTCAGCACCCTGATTTCTCTCCTGAATCCTTCAGAATTTTGGCCTACGGTGAT
GACGTGATCTATGGGTGCGACCCACCCATCCATCCGTCATTCATCAAGGAG
