# Supplementary material for: Molecular Detection and Genetic Characterization of Potential Zoonotic Swine Enteric Viruses in Northern China
Source: Pathogens. 2022 Mar 30;11(4):417. doi: 10.3390/pathogens11040417 (PMC9031704; doi:10.3390/pathogens11040417)
Supplement: Supplementary file 1 [file pathogens-11-00417-s001.zip › PSaV.pdf]

>sapo-1
TGACAATTGAGACCCGTCCGGGCCAGGACTTTGGATTCACCCTGCTCAAGCCCCCAAACC
AAACCATGGAGGTGGGATTCGACCCCAGGTCACTCCTGCCCCGCACGGCAAGAACGCTTC
GGGGGAACAGGTTTGGTAGACCTATTACAGCTGTGGTCATAGTGGGTTTGGCGCAACAAA
TCAACAGGCACTTTTCAGCTGACGGCACTACACTTGGCTGGTCCACGGCCCCAATTGCTC
CGTGTGTGGCACGTGTCAACGGAAAGTACACTGGCACGAATGGCATGGCAGTATTTCAGC
TTCAACCCCTTAGCAATGGGCCCCTTTACCCCAACATTGTCAACCATTACCCAGATGTGG
CCGCATCAACAATACTCAGTGACAGGACCTCCATTGCTGACAACCTGACGTGTGGTGGGG
GGCCTATGGTGGTTTTTGATGATCAGGGTGATGTGACTGAGACTGTGGCCTACCAGATGA
GATTCATAGCCTCACACGCCACTTCCCAAAATCCCACACTTGTTGACAAAATCAATGCAA
CAACAATGGCTGTGTGCAGTTTTGGCAATTCACGGGCAGATCTCGGCCAATCCCAGCTTA
ACGTGGGCATTGAACTGACTTACACCTGTGGTGAGACACCGATCAACGGGAATGTCACTC
CGTTCATGGATCGCCAGTACACATTTGGCGCACAGGG
>sapo-2|SPF
TAACAGTAGAGACCCGTCCCGGTGCAGACTTTGGGTTCACCCTGCTCAAGCCTCCAAACC
AAACCATGGAGGTGGGACTTGACCCCAGGTCGCTCCTGCCCCGCACTGCAAGAACACTGC
GGGGGAACAGGTTTGGCAGGCCCATCAGATCTGTGCTCATAGTGGGTTTGGCACAACAAA
TTAATAGGCACTTTTCAGCAGAGGGCACCACACTTGGTTGGTCCACGGCCCCAATTGGCC
CCTGTGTGGGCCGCATTAACACAAAGTACACTGGCACTGGGGGCAAGGTGGTGGCTCAAC
TGCTGCCTTTGAGTAACGGGCCCCTTTACCCAAATATCATCAACCACTACCCAGATGTGG
CTGCATCAACAATACTCAGTGGAGGGTCTAGCATAACTAACGACATGACGTGTGGGGGAG
GACCTATGGTGCTTTTCAACAATGTGGGCGATGTAGTGGAGACCATCTCCTACCAAATGA
GGTTCATAGCCTCACAGGCCACATCTCAAAACACCACACTCATCGATAAGATCAATGCAA
CATCAATGTCAGTGGTCAGTTTTGACAACTCCCGGGGTGACTTCCCCCAATCGGAACACA
ATGTGGGTATTGAGTTGACCTACACATGTGGTCCCACACCAATCAACGGGAACGTCACCC
AGTTCATGGACCGCCAATATACCTTTGGCGCACAGGG
>sapo-3|SPF
TAACAGTAGAGACCCGTCCCGGTGCAGACTTTGGGTTCACCCTGCTCAAGCCTCCAAACC
AAACCATGGAGGTGGGACTTGACCCCAGGTCGCTCCTGCCCCGCACTGCAAGAACACTGC
GGGGGAACAGGTTTGGCAGGCCCATCAGATCTGTGCTCATAGTGGGTTTGGCACAACAAA
TTAATAGGCACTTTTCAGCAGAGGGCACCACACTTGGTTGGTCCACGGCCCCAATTGGCC
CCTGTGTGGGCCGCATTAACACAAAGTACACTGGCACTGGGGGCAAGGTGGTGGCTCAAC
TGCTGCCTTTGAGTAACGGGCCCCTTTACCCAAATATCATCAACCACTACCCAGATGTGG
CTGCATCAACAATACTCAGTGGAGGGTCTAGCATAACTAACGACATGACGTGTGGGGGAG
GACCTATGGTGCTTTTCAACAATGTGGGCGATGTAGTGGAGACCATCTCCTACCAAATGA
GGTTCATAGCCTCACAGGCCACATCTCAAAACACCACACTCATCGATAAGATCAATGCAA
CATCAATGTCAGTGGTCAGTTTTGACAACTCCCGGGGTGACTTCCCCCAATCGGAACACA
ATGTGGGTATTGAGTTGACCTACACATGTGGTCCCACACCAATCAACGGGAACGTCACCC
AGTTCATGGACCGCCAATATACCTTTGGCGCACAGGG
>sapo-4|SPF
TAACAGTAGAGACCCGTCCCGGTGCAGACTTTGGGTTCACCCTGCTCAAGCCTCCAAACC
AAACCATGGAGGTGGGACTTGACCCCAGGTCGCTCCTGCCCCGCACTGCAAGAACACTGC
GGGGGAACAGGTTTGGCAGGCCCATCAGATCTGTGCTCATAGTGGGTTTGGCACAACAAA
TTAATAGGCACTTTTCAGCAGAGGGCACCACACTTGGTTGGTCCACGGCCCCAATTGGCC
CCTGTGTGGGCCGCATTAACACAAAGTACACTGGCACTGGGGGCAAGGTGGTGGCTCAAC
TGCTGCCTTTGAGTAACGGGCCCCTTTACCCAAATATCATCAACCACTACCCAGATGTGG
CTGCATCAACAATACTCAGTGGAGGGTCTAGCATAACTAACGACATGACGTGTGGGGGAG
GACCTATGGTGCTTTTCAACAATGTGGGCGATGTAGTGGAGACCATCTCCTACCAAATGA
GGTTCATAGCCTCACAGGCCACATCTCAAAACACCACACTCATCGATAAGATCAATGCAA
CATCAATGTCAGTGGTCAGTTTTGACAACTCCCGGGGTGACTTCCCCCAATCGGAACACA
ATGTGGGTATTGAGTTGACCTACACATGTGGTCCCACACCAATCAACGGGAACGTCACCC
AGTTCATGGACCGCCAATATACCTTTGGCGCACAGGG
>sapo-5|SPF

TGACAGTCGAGACCCGTCCCGGCGCAGACTTTGGGTTTACCCTGCTCAAGCCTCCAAACC
AAACCATGGAGGTGGGACTCGACCCCAGGTCGCTCCTGCCCCGCACCGCAAGAACACTGC
GGGGGAACAGGTTTGGCAGGCCCATCAGATCTGTGATCATAGTAGGTTTGGCACAACAAA
TCAACAGGCATTTTTCCGCAGAGGGTACCACACTTGGTTGGTCCACGGCCCCAATTGGCC
CCTGTGTAGGCCGCATCAACACCAAGTACACTGGTAATGCGGGCAAGGTGGTAGCTCAAT
TGCTACCTTTGAGCAATGGGCCCCTTTACCCAAACATCATCAACCACTATCCAGATGTGG
CTGCATCAACAATGCTCAGTGGAGGGTCTAGCATAACTACTGACATGACGTGTGGGGGAG
GGCCCATGGTGCTCTTCAACGATGTGGGTGATGTGGTGGAGACCGTCTCCTACCAAATGA
GGTTCATAGCCTCACAGGCCACATCTCAAAGCACCACACTCATCGACAAGATCAACGCAA
CATCAATGTCAGTGGTCAGCTTTGACAACTCCCGGAATGACTTCCCTCAATCAAATGACA
ATGTGGGCATTGAGTTAACCTACACTTGTGGCAACACACCGATCAATGGGAATGTCACCC
AGTTCATGGACCGCCAATATACTTTTGGCGCACAGGG
>sapo-6
TGACAGTCGAGACCCGTCCCGGCGCAGACTTTGGGTTTACCCTGCTCAAGCCTCCAAACC
AAACCATGGAGGTGGGACTCGACCCCAGGTCGCTCCTGCCCCGCACCGCAAGAACACTGC
GGGGGAACAGGTTTGGCAGGCCCATCAGATCTGTGATCATAGTAGGTTTGGCACAACAAA
TCAACAGGCATTTTTCCGCAGAGGGTACCACACTTGGTTGGTCCACGGCCCCAATTGGCC
CCTGTGTAGGCCGCATCAACACCAAGTACACTGGTAATGCGGGCAAGGTGGTAGCTCAAT
TGCTACCTTTGAGCAATGGGCCCCTTTACCCAAACATCATCAACCACTATCCAGATGTGG
CTGCATCAACAATGCTCAGTGGAGGGTCTAGCATAACTACTGACATGACGTGTGGGGGAG
GGCCCATGGTGCTCTTCAACGATGTGGGTGATGTGGTGGAGACCGTCTCCTACCAAATGA
GGTTCATAGCCTCACAGGCCACATCTCAAAGCACCACACTCATCGACAAGATCAACGCAA
CATCAATGTCAGTGGTCAGCTTTGACAACTCCCGGAATGACTTCCCTCAATCAAATGACA
ATGTGGGCATTGAGTTAACCTACACTTGTGGCAACACACCGATCAATGGGAATGTCACCC
AGTTCATGGACCGCCAATATACTTTTGGCGCACAGGG
>sapo-7
TGACAGTCGAGACCCGTCCCGGCGCAGACTTTGGGTTTACCCTGCTCAAGCCTCCAAACC
AAACCATGGAGGTGGGACTCGACCCCAGGTCGCTCCTGCCCCGCACCGCAAGAACACTGC
GGGGGAACAGGTTTGGCAGGCCCATCAGATCTGTGATCATAGTAGGTTTGGCACAACAAA
TCAACAGGCATTTTTCCGCAGAGGGTACCACACTTGGTTGGTCCACGGCCCCAATTGGCC
CCTGTGTAGGCCGCATCAACACCAAGTACACTGGTAATGCGGGCAAGGTGGTAGCTCAAT
TGCTACCTTTGAGCAATGGGCCCCTTTACCCAAACATCATCAACCACTATCCAGATGTGG
CTGCATCAACAATGCTCAGTGGAGGGTCTAGCATAACTACTGACATGACGTGTGGGGGAG
GGCCCATGGTGCTCTTCAACGATGTGGGTGATGTGGTGGAGACCGTCTCCTACCAAATGA
GGTTCATAGCCTCACAGGCCACATCTCAAAGCACCACACTCATCGACAAGATCAACGCAA
CATCAATGTCAGTGGTCAGCTTTGACAACTCCCGGAATGACTTCCCTCAATCAAATGACA
ATGTGGGCATTGAGTTAACCTACACTTGTGGCAACACACCGATCAATGGGAATGTCACCC
AGTTCATGGACCGCCAATATACTTTTGGCGCACAGGG
>sapo-8
TGACAGTCGAGACCCGTCCCGGCGCAGACTTTGGGTTTACCCTGCTCAAGCCTCCAAACC
AAACCATGGAGGTGGGACTCGACCCCAGGTCGCTCCTGCCCCGCACCGCAAGAACACTGC
GGGGGAACAGGTTTGGCAGGCCCATCAGATCTGTGATCATAGTAGGTTTGGCACAACAAA
TCAACAGGCATTTTTCCGCAGAGGGTACCACACTTGGTTGGTCCACGGCCCCAATTGGCC
CCTGTGTAGGCCGCATCAACACCAAGTACACTGGTAATGCGGGCAAGGTGGTAGCTCAAT
TGCTACCTTTGAGCAATGGGCCCCTTTACCCAAACATCATCAACCACTATCCAGATGTGG
CTGCATCAACAATGCTCAGTGGAGGGTCTAGCATAACTACTGACATGACGTGTGGGGGAG
GGCCCATGGTGCTCTTCAACGATGTGGGTGATGTGGTGGAGGCCGTCTCCTACCAAATGA
GGTTCATAGCCTCACAGGCCACATCTCAAAGCACCACACTCATCGACAAGATCAACGCAA
CATCAATGTCAGTGGTCAGCTTTGACAACTCCCGGAATGACTTCCCTCAATCAAATGACA
ATGTGGGCATTGAGTTAACCTACACTTGTGGCAACACACCGATCAATGGGAATGTCACCC
AGTTCATGGACCGCCAATATACTTTTGGCGCACAGGG
>sapo-9
TGACAGTCGAGACCCGTCCCGGCGCAGACTTTGGGTTTACCCTGCTCAAGCCTCCAAACC

AAACCATGGAGGTGGGACTCGACCCCAGGTCGCTCCTGCCCCGCACCGCAAGAACACTGC
GGGGGAACAGGTTTGGCAGGCCCATCAGATCTGTGATCATAGTAGGTTTGGCACAACAAA
TCAACAGGCATTTTTCCGCAGAGGGTACCACACTTGGTTGGTCCACGGCCCCAATTGGCC
CCTGTGTAGGCCGCATCAACACCAAGTACACTGGTAATGCGGGCAAGGTGGTAGCTCAAT
TGCTACCTTTGAGCAATGGGCCCCTTTACCCAAACATCATCAACCACTATCCAGATGTGG
CTGCATCAACAATGCTCAGTGGAGGGTCTAGCATAACTACTGACATGACGTGTGGGGGAG
GGCCCATGGTGCTCTTCAACGATGTGGGTGATGTGGTGGAGACCGTCTCCTACCAAATGA
GGTTCATAGCCTCACAGGCCACATCTCAAAGCACCACACTCATCGACAAGATCAACGCAA
CATCAATGTCAGTGGTCAGCTTTGACAACTCCCGGAATGACTTCCCTCAATCAAATGACA
ATGTGGGCATTGAGTTAACCTACACTTGTGGCAACACACCGATCAATGGGAATGTCACCC
AGTTCATGGACCGCCAATATACTTTTGGCGCACAGGG
>sapo-10
TGACAGTCGAGACCCGTCCCGGCGCAGACTTTGGGTTTACCCTGCTCAAGCCTCCAAACC
AAACCATGGAGGTGGGACTCGACCCCAGGTCGCTCCTGCCCCGCACCGCAAGAACACTGC
GGGGGAACAGGTTTGGCAGGCCCATCAGATCTGTGATCATAGTAGGTTTGGCACAACAAA
TCAACAGGCATTTTTCCGCAGAGGGTACCACACTTGGTTGGTCCACGGCCCCAATTGGCC
CCTGTGTAGGCCGCATCAACACCAAGTACACTGGTAATGCGGGCAAGGTGGTAGCTCAAT
TGCTACCTTTGAGCAATGGGCCCCTTTACCCAAACATCATCAACCACTATCCAGATGTGG
CTGCATCAACAATGCTCAGTGGAGGGTCTAGCATAACTACTGACATGACGTGTGGGGGAG
GGCCCATGGTGCTCTTCAACGATGTGGGTGATGTGGTGGAGACCGTCTCCTACCAAATGA
GGTTCATAGCCTCACAGGCCACATCTCAAAGCACCACACTCATCGACAAGATCAACGCAA
CATCAATGTCAGTGGTCAGCTTTGACAACTCCCGGAATGACTTCCCTCAATCAAATGACA
ATGTGGGCATTGAGTTAACCTACACTTGTGGCAACACACCGATCAATGGGAATGTCACCC
AGTTCATGGACCGCCAATATACTTTTGGCGCACAGGG
>sapo-11
TGACAGTCGAGACCCGTCCCGGCGCAGACTTTGGGTTTACCCTGCTCAAGCCTCCAAACC
AAACCATGGAGGTGGGACTCGACCCCAGGTCGCTCCTGCCCCGCACCGCAAGAACACTGC
GGGGGAACAGGTTTGGCAGGCCCATCAGATCTGTGATCATAGTAGGTTTGGCACAACAAA
TCAACAGGCATTTTTCCGCAGAGGGTACCACACTTGGTTGGTCCACGGCCCCAATTGGCC
CCTGTGTAGGCCGCATCAACACCAAGTACACTGGTAATGCGGGCAAGGTGGTAGCTCAAT
TGCTACCTTTGAGCAATGGGCCCCTTTACCCAAACATCATCAACCACTATCCAGATGTGG
CTGCATCAACAATGCTCAGTGGAGGGTCTAGCATAACTACTGACATGACGTGTGGGGGAG
GGCCCATGGTGCTCTTCAACGATGTGGGTGATGTGGTGGAGACCGTCTCCTACCAAATGA
GGTTCATAGCCTCACAGGCCACATCTCAAAGCACCACACTCATCGACAAGATCAACGCAA
CATCAATGTCAGTGGTCAGCTTTGACAACTCCCGGAATGACTTCCCTCAATCAAATGACA
ATGTGGGCATTGAGTTAACCTACACTTGTGGCAACACACCGATCAATGGGAATGTCACCC
AGTTCATGGACCGCCAATATACTTTTGGCGCACAGGG
>sapo-12
TGACAGTCGAGACCCGTCCCGGCGCAGACTTTGGGTTTACCCTGCTCAAGCCTCCAAACC
AAACCATGGAGGTGGGACTCGACCCCAGGTCGCTCCTGCCCCGCACCGCAAGAACACTGC
GGGGGAACAGGTTTGGCAGGCCCATCAGATCTGTGATCATAGTAGGTTTGGCACAACAAA
TCAACAGGCATTTTTCCGCAGAGGGTACCACACTTGGTTGGTCCACGGCCCCAATTGGCC
CCTGTGTAGGCCGCATCAACACCAAGTACACTGGTAATGCGGGCAAGGTGGTAGCTCAAT
TGCTACCTTTGAGCAATGGGCCCCTTTACCCAAACATCATCAACCACTATCCAGATGTGG
CTGCATCAACAATGCTCAGTGGAGGGTCTAGCATAACTACTGACATGACGTGTGGGGGAG
GGCCCATGGTGCTCTTCAACGATGTGGGTGATGTGGTGGAGACCGTCTCCTACCAAATGA
GGTTCATAGCCTCACAGGCCACATCTCAAAGCACCACACTCATCGACAAGATCAACGCAA
CATCAATGTCAGTGGTCAGCTTTGACAACTCCCGGAATGACTTCCCTCAATCAAATGACA
ATGTGGGCATTGAGTTAACCTACACTTGTGGCAACACACCGATCAATGGGAATGTCACCC
AGTTCATGGACCGCCAATATACTTTTGGCGCACAGGG
>sapo-13
TGACAGTCGAGACCCGTCCCGGCGCAGACTTTGGGTTTACCCTGCTCAAGCCTCCAAACC
AAACCATGGAGGTGGGACTCGACCCCAGGTCGCTCCTGCCCCGCACCGCAAGAACACTGC

GGGGGAACAGGTTTGGCAGGCCCATCAGATCTGTGATCATAGTAGGTTTGGCACAACAAA
TCAACAGGCATTTTTCCGCAGAGGGTACCACACTTGGTTGGTCCACGGCCCCAATTGGCC
CCTGTGTAGGCCGCATCAACACCAAGTACACTGGTAATGCGGGCAAGGTGGTAGCTCAAT
TGCTACCTTTGAGCAATGGGCCCCTTTACCCAAACATCATCAACCACTATCCAGATGTGG
CTGCATCAACAATGCTCAGTGGAGGGTCTAGCATAACTACTGACATGACGTGTGGGGGAG
GGCCCATGGTGCTCTTCAACGATGTGGGTGATGTGGTGGAGACCGTCTCCTACCAAATGA
GGTTCATAGCCTCACAGGCCACATCTCAAAGCACCACACTCATCGACAAGATCAACGCAA
CATCAATGTCAGTGGTCAGCTTTGACAACTCCCGGAATGACTTCCCTCAATCAAATGACA
ATGTGGGCATTGAGTTAACCTACACTTGTGGCAACACACCGATCAATGGGAATGTCACCC
AGTTCATGGACCGCCAATATACTTTTGGCGCACAGGG
>sapo-14
TGACAGTCGAGACCCGTCCCGGCGCAGACTTTGGGTTTACCCTGCTCAAGCCTCCAAACC
AAACCATGGAGGTGGGACTCGACCCCAGGTCGCTCCTGCCCCGCACCGCAAGAACACTGC
GGGGGAACAGGTTTGGCAGGCCCATCAGATCTGTGATCATAGTAGGTTTGGCACAACAAA
TCAACAGGCATTTTTCCGCAGAGGGTACCACACTTGGTTGGTCCACGGCCCCAATTGGCC
CCTGTGTAGGCCGCATCAACACCAAGTACACTGGTAATGCGGGCAAGGTGGTAGCTCAAT
TGCTACCTTTGAGCAATGGGCCCCTTTACCCAAACATCATCAACCACTATCCAGATGTGG
CTGCATCAACAATGCTCAGTGGAGGGTCTAGCATAACTACTGACATGACGTGTGGGGGAG
GGCCCATGGTGCTCTTCAACGATGTGGGTGATGTGGTGGAGACCGTCTCCTACCAAATGA
GGTTCATAGCCTCACAGGCCACATCTCAAAGCACCACACTCATCGACAAGATCAACGCAA
CATCAATGTCAGTGGTCAGCTTTGACAACTCCCGGAATGACTTCCCTCAATCAAATGACA
ATGTGGGCATTGAGTTAACCTACACTTGTGGCAACACACCGATCAATGGGAATGTCACCC
AGTTCATGGACCGCCAATATACTTTTGGCGCACAGGG
>sapo-15
TGACAGTCGAGACCCGTCCCGGCGCAGACTTTGGGTTTACCCTGCTCAAGCCTCCAAACC
AAACCATGGAGGTGGGACTCGACCCCAGGTCGCTCCTGCCCCGCACCGCAAGAACACTGC
GGGGGAACAGGTTTGGCAGGCCCATCAGATCTGTGATCATAGTAGGTTTGGCACAACAAA
TCAACAGGCATTTTTCCGCAGAGGGTACCACACTTGGTTGGTCCACGGCCCCAATTGGCC
CCTGTGTAGGCCGCATCAACACCAAGTACACTGGTAATGCGGGCAAGGTGGTAGCTCAAT
TGCTACCTTTGAGCAATGGGCCCCTTTACCCAAACATCATCAACCACTATCCAGATGTGG
CTGCATCAACAATGCTCAGTGGAGGGTCTAGCATAACTACTGACATGACGTGTGGGGGAG
GGCCCATGGTGCTCTTCAACGATGTGGGTGATGTGGTGGAGACCGTCTCCTACCAAATGA
GGTTCATAGCCTCACAGGCCACATCTCAAAGCACCACACTCATCGACAAGATCAACGCAA
CATCAATGTCAGTGGTCAGCTTTGACAACTCCCGGAATGACTTCCCTCAATCAAATGACA
ATGTGGGCATTGAGTTAACCTACACTTGTGGCAACACACCGATCAATGGGAATGTCACCC
AGTTCATGGACCGCCAATATACTTTTGGCGCACAGGG
>sapo-16
GAAGAATTGAGACCCGTCCGGGCCAGGACTTTGGATTCACCCTGCTCAAGCCCCCAAACC
AAACCATGGAGGTGGGATTCGACCCCAGGTCACTCCTGCCCCGCACGGCAAGAACGCTTC
GGGGGAACAGGTTTGGTAGACCTATTACAGCTGTGGTCATAGTGGGTTTGGCGCAACAAA
TCAACAGGCACTTTTCAGCTGACGGCACTACACTTGGCTGGTCCACGGCCCCAATTGCTC
CGTGTGTGGCACGTGTCAACGGAAAGTACACTGGCACGAATGGCATGGCAGTATTTCAGC
TTCAACCCCTTAGCAATGGGCCCCTTTACCCCAACATTGTCAACCATTACCCAGATGTGG
CCGCATCAACAATACTCAGTGACAGGACCTCCATTGCTGACAACCTGACGTGTGGTGGGG
GGCCTATGGTGGTTTTTGATGATCAGGGTGATGTGACTGAGACTGTGGCCTACCAGATGA
GATTCATAGCCTCACACGCCACTTCCCAAAATCCCACACTTGTTGACAAAATCAATGCAA
CAACAATGGCTGTGTGCAGTTTTGGCAATTCACGGGCAGATCTCGGCCAATCCCAGCTTA
ACGTGGGCATTGAACTGACTTACACCTGTGGTGAGACACCGATCAACGGGAATGTCACTC
CGTTCATGGATCGCCAGTACACATTTGGCGCACAGGG
>sapo-17
GGTGAATTGAGACCCGTCCGGGCCAGGACTTTGGATTCACCCTGCTCAAGCCCCCAAACC
AAACCATGGAGGTGGGATTCGACCCCAGGTCACTCCTGCCCCGCACGGCAAGAACGCTTC
GGGGGAACAGGTTTGGTAGACCTATTACAGCTGTGGTCATCGTGGGTTTGGCGCAACAAA

TCAACAGGCACTTTTCAGCTGACGGCACTACACTTGGCTGGTCCACGGCCCCAATTGCTC
CGTGTGTGGCACGTGTCAACGGAAAGTACACTGGCACGAATGGCATGGCAGTATTTCAGC
TTCAACCCCTTAGCAATGGGCCCCTTTACCCCAACATTGTCAACCATTACCCAGATGTGG
CCGCATCAACAATACTCAGTGACAGGACCTCCATTGCTGACAACCTGACGTGTGGTGGGG
GGCCTATGGTGGTTTTTGATGATCAGGGTGATGTGACTGAGACTGTGGCCTACCAGATGA
GATTCATAGCCTCACACGCCACTTCCCAAAATCCCACACTTGTTGACAAAATCAATGCAA
CAACAATGGCTGTGTGCAGTTTTGGCAATTCACGGGCAGATCTCGGCCAATCCCAGCTTA
ACGTGGGCATTGAACTGACTTACACCTGTGGTGAGACACCGATCAACGGGAATGTCACTC
CGTTCATGGATCGCCAGTACACATTTGGCGCACAGGG
>sapo-18
TGTGAATTGAGACCCGTCCGGGCCAGGACTTTGGATTCACCCTGCTCAAGCCCCCAAACC
AAACCATGGAGGTGGGATTCGACCCCAGGTCACTCCTGCCCCGCACGGCAAGAACGCTTC
GGGGGAACAGGTTTGGTAGACCTATTACAGCTGTGGTCACAGTGGGTTTGGCGCAACAAA
TCAACAGGCACTTTTCAGCTGACGGCACTACACTTGGCTGGTCCACGGCCCCAATTGCTC
CGTGTGTGGCACGTGTCAACGGAAAGTACACTGGCACGAATGGCATGGCAGTATTTCAGC
TTCAACCCCTTAGCAATGGGCCCCTTTACCCCAACATTGTCAACCATTACCCAGATGTGG
CCGCATCAACAATACTCAGTGACAGGACCTCCATTGCTGACAACCTGACGTGTGGTGGGG
GGCCTATGGTGGTTTTTGATGATCAGGGTGATGTGACTGAGACTGTGGCCTACCAGATGA
GATTCATAGCCTCACACGCCACTTCCCAAAATCCCACACTTGTTGACAAAATCAATGCAA
CAACAATGGCTGTGTGCAGTTTTGGCAATTCACGGGCAGATCTCGGCCAATCCCAGCTTA
ACGTGGGCATTGAACTGACTTACACCTGTGGTGAGACACCGATCAACGGGAATGTCACTC
CGTTCATGGATCGCCAGTACACATTTGGCGCACAGGG
>sapo-19
TGTGAATTGAGACCCGTCCGGGCCAGGACTTTGGATTCACCCTGCTCAAGCCCCCAAACC
AAACCATGGAGGTGGGATTCGACCCCAGGTCACTCCTGCCCCGCACGGCAAGAACGCTTC
GGGGGAACAGGTTTGGTAGACCTATTACAGCTGTGGTCACAGTGGGTTTGGCGCAACAAA
TCAACAGGCACTTTTCAGCTGACGGCACTACACTTGGCTGGTCCACGGCCCCAATTGCTC
CGTGTGTGGCACGTGTCAACGGAAAGTACACTGGCACGAATGGCATGGCAGTATTTCAGC
TTCAACCCCTTAGCAATGGGCCCCTTTACCCCAACATTGTCAACCATTACCCAGATGTGG
CCGCATCAACAATACTCAGTGACAGGACCTCCATTGCTGACAACCTGACGTGTGGTGGGG
GGCCTATGGTGGTTTTTGATGATCAGGGTGATGTGACTGAGACTGTGGCCTACCAGATGA
GATTCATAGCCTCACACGCCACTTCCCAAAATCCCACACTTGTTGACAAAATCAATGCAA
CAACAATGGCTGTGTGCAGTTTTGGCAATTCACGGGCAGATCTCGGCCAATCCCAGCTTA
ACGTGGGCATTGAACTGACTTACACCTGTGGTGAGACACCGATCAACGGGAATGTCACTC
CGTTCATGGATCGCCAGTACACATTTGGCGCACAGGG
>sapo-20|SPF
TAACAGTAGAGACCCGTCCCGGTGCAGACTTTGGGTTCACCCTGCTCAAGCCTCCAAACC
AAACCATGGAGGTGGGACTTGACCCCAGGTCGCTCCTGCCCCGCACTGCAAGAACACTGC
GGGGGAACAGGTTTGGCAGGCCCATCAGATCTGTGCTCATAGTGGGTTTGGCACAACAAA
TTAATAGGCACTTTTCAGCAGAGGGCACCACACTTGGTTGGTCCACGGCCCCAATTGGCC
CCTGTGTGGGCCGCATTAACACAAAGTACACTGGCACTGGGGGCAAGGTGGTGGCTCAAC
TGCTGCCTTTGAGTAACGGGCCCCTTTACCCAAATATCATCAACCACTACCCAGATGTGG
CTGCATCAACAATACTCAGTGGAGGGTCTAGCATAACTAACGACATGACGTGTGGGGGAG
GACCTATGGTGCTTTTCAACAATGTGGGCGATGTAGTGGAGACCATCTCCTACCAAATGA
GGTTCATAGCCTCACAGGCCACATCTCAAAACACCACACTCATCGATAAGATCAATGCAA
CATCAATGTCAGTGGTCAGTTTTGACAACTCCCGGGGTGACTTCCCCCAATCGGAACACA
ATGTGGGTATTGAGTTGACCTACACATGTGGTCCCACACCAATCAACGGGAACGTCACCC
AGTTCATGGACCGCCAATATACCTTTGGCGCACAGGG
